# Integrating a microRNA signature as a liquid biopsy-based tool for the early diagnosis and prediction of potential therapeutic targets in pancreatic cancer

**DOI:** 10.1038/s41416-023-02488-4

**Published:** 2023-11-10

**Authors:** Wenjie Shi, Thomas Wartmann, Sara Accuffi, Sara Al-Madhi, Aristotelis Perrakis, Christoph Kahlert, Alexander Link, Marino Venerito, Verena Keitel-Anselmino, Christiane Bruns, Roland S. Croner, Yue Zhao, Ulf D. Kahlert

**Affiliations:** 1 Molecular and Experimental Surgery, Faculty of Medicine and University Hospital Magdeburg, Department of General-, Visceral-, Vascular- and Transplant- Surgery, University of Magdeburg, Magdeburg, Germany; 2 Department of General, Visceral and Transplantation Surgery, Heidelberg University Hospital, Heidelberg, Germany; 3 Department of Gastroenterology, Hepatology and Infectious Diseases, Otto-von-Guericke University Hospital Magdeburg, 39120 Magdeburg, Germany; 4 Faculty of Medicine and University Hospital Cologne, Department of General, Visceral and Cancer Surgery, University of Cologne, Cologne, Germany

**Keywords:** Cancer screening, Diagnostic markers

## Abstract

**Introduction:**

Pancreatic cancer is a highly aggressive cancer, and early diagnosis significantly improves patient prognosis due to the early implementation of curative-intent surgery. Our study aimed to implement machine-learning algorithms to aid in early pancreatic cancer diagnosis based on minimally invasive liquid biopsies.

**Materials and methods:**

The analysis data were derived from nine public pancreatic cancer miRNA datasets and two sequencing datasets from 26 pancreatic cancer patients treated in our medical center, featuring small RNAseq data for patient-matched tumor and non-tumor samples and serum. Upon batch-effect removal, systematic analyses for differences between paired tissue and serum samples were performed. The robust rank aggregation (RRA) algorithm was used to reveal feature markers that were co-expressed by both sample types. The repeatability and real-world significance of the enriched markers were then determined by validating their expression in our patients’ serum. The top candidate markers were used to assess the accuracy of predicting pancreatic cancer through four machine learning methods. Notably, these markers were also applied for the identification of pancreatic cancer and pancreatitis. Finally, we explored the clinical prognostic value, candidate targets and predict possible regulatory cell biology mechanisms involved.

**Results:**

Our multicenter analysis identified hsa-miR-1246, hsa-miR-205-5p, and hsa-miR-191-5p as promising candidate serum biomarkers to identify pancreatic cancer. In the test dataset, the accuracy values of the prediction model applied via four methods were 94.4%, 84.9%, 82.3%, and 83.3%, respectively. In the real-world study, the accuracy values of this miRNA signatures were 82.3%, 83.5%, 79.0%, and 82.2. Moreover, elevated levels of these miRNAs were significant indicators of advanced disease stage and allowed the discrimination of pancreatitis from pancreatic cancer with an accuracy rate of 91.5%. Elevated expression of hsa-miR-205-5p, a previously undescribed blood marker for pancreatic cancer, is associated with negative clinical outcomes in patients.

**Conclusion:**

A panel of three miRNAs was developed with satisfactory statistical and computational performance in real-world data. Circulating hsa-miRNA 205-5p serum levels serve as a minimally invasive, early detection tool for pancreatic cancer diagnosis and disease staging and might help monitor therapy success.

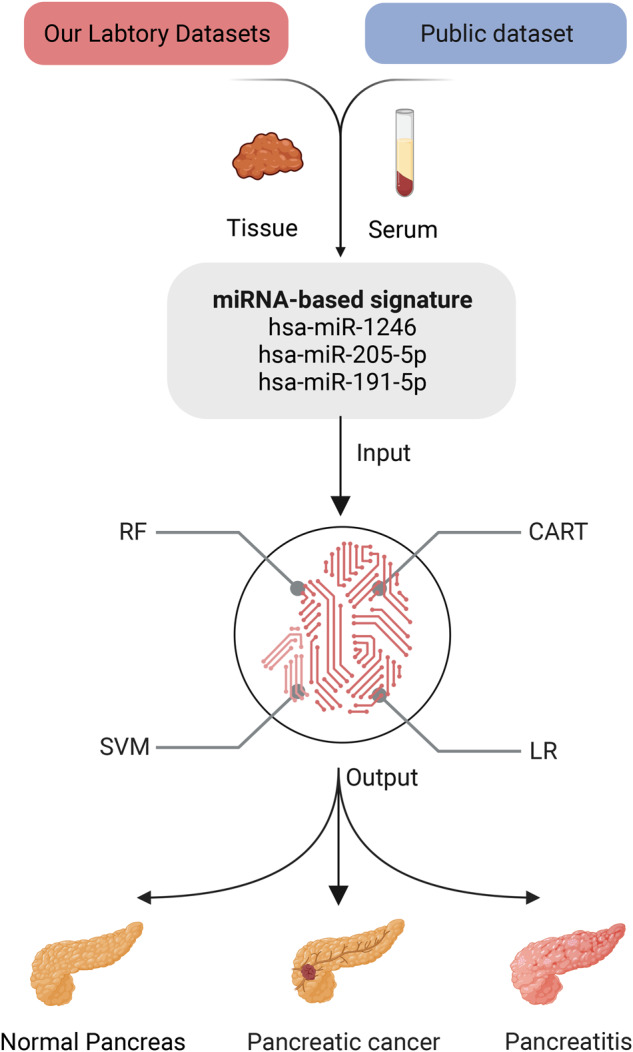

## Introduction

Pancreatic cancer (PC) is a highly aggressive cancer with a 5-year survival rate of approximately 5–10% upon diagnosis under the standard of care therapy [1]. Regrettably, the incidence of PC is on the rise [2, 3]. The high mortality rate of PC is mainly due to the fact that only a minority of patients (11%) are diagnosed with localized disease, while 52% of PC cases are diagnosed in the late stage, at which point few patients can receive curative intervention [4, 5]. Therefore, early detection of PC plays a critical role in improving overall outcomes.

Minimally invasive methods such as liquid biopsy-based cancer biomarker evaluation promise hope for early-stage PC detection, resolution of unclear clinical imaging-detected lesions or prevention of cancer progression in patients with minor pancreatic abnormalities [6]. The widely applied serological marker for pancreatic tumors in clinical practice is Carbohydrate Antigen 19-9 (CA 19-9), but its unequivocal power in terms of specificity, sensitivity, and predictive value for outcomes such as overall survival (OS) has been challenged in recent studies [7, 8]. Moreover, in 10% of the population, the lack of Lewis antigens precludes the CA 19-9 level from being informative [9, 10]. Other recently discovered blood biomarkers, such as circulating tumor cells (CTCs), exosomes [11], circulating tumor DNA (ctDNA), or cell-free DNA (cfDNA), have shown promising insights, but there are major obstacles regarding their clinical applicability and verification as biomarkers. For instance, one of the major challenges of ctDNA/cfDNA application in PC is the extremely low levels of peripheral DNA, which can have a significant impact on sensitivity [12], especially in the case of early-stage disease. In addition, clinically relevant and repeatable assays involving the use of DNA methylation markers in ctDNA have emerged and been shown to provide information on disease manifestation including minimal residual disease status of many cancers. Although very powerful and promising, sample preparation for DNA methylation analysis requires many resources especially compared to those required for free RNA detection, which limits stringent clinical application of this technology in routine use [13, 14].

In contrast, mature miRNAs are highly stable in body fluids and exhibit exceptional specificity across different cancer types, making them as promising non-invasive biomarkers for cancer [15, 16]. Screening of tumor miRNA markers in body fluids holds great clinical value in assisting in the early diagnosis of disease and monitoring therapy success longitudinally. For liquid biopsy assays, the fraction of the sample needed to serve as a template for the serum, plasma and whole blood content of the same donor has been shown to vary significantly [17]. In this study, we focused on the serum of patients diagnosed with PC.

In addition to the discussed blood-based biomarkers, strategies employing artificial intelligence are being developed in parallel to improve the efficacy of early screening, and this approach is being highly emphasized [6, 18, 19]. We propose the integration of machine learning algorithms with real-world and public database analyses for the early screening of circulating miRNAs in PC. We propose that our results might assist the further development of early detection strategies by using minimally invasive screening tools for PC diagnosis and prognosis.

## Materials and methods

### Patient cohort enrollment and miRNA sequencing

**Fig. 1:**
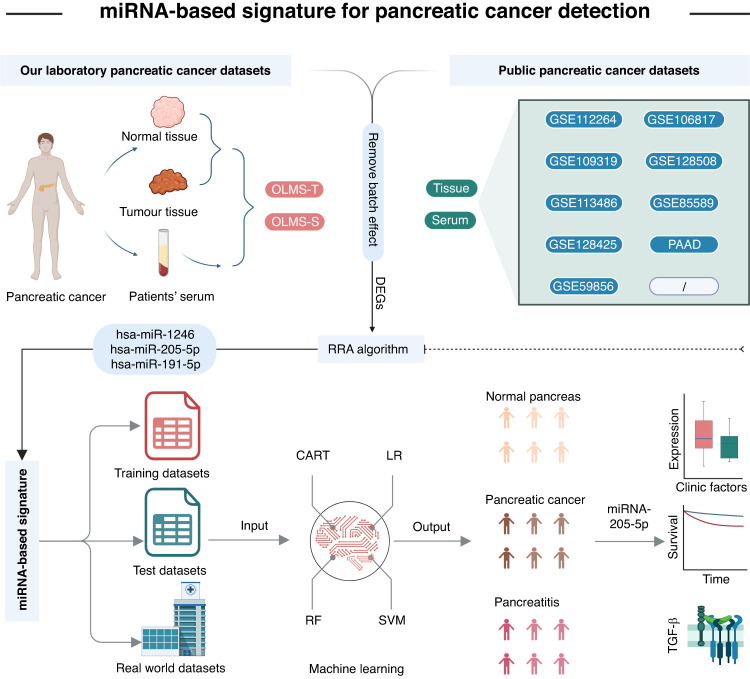
Schematic presentation of the workflow of our study. Own laboratory miRNA sequencing (OLMS) from patient-matched bio samples of from tumor (T), healthy pancreas tissue (HT) and serum (S) presents the real-world dataset (upper left panel). Computational assessment of this data together with nine publically available, context-related dataset featuring tissue and serum RNA-sequencing data (upper right panel) identified a microRNA candidate signature for identifying pancreatic cancer in patient serum. With the use of four machine learning algorithms, the specificity, sensitivity of those and selected candidate microRNA 205-5p were tested. Our data reveal a new possible strategy to identify pancreatic cancer, predict the clinical course of the patient and verify tumor resection completeness (lower panel).

We collected patient serum samples before the onset of any therapy (including neoadjuvant chemotherapy and surgery). The PC tissues and paired para-cancerous tissue, defined as healthy tissues (HT), were collected from operation specimens as defined by the existence of tumor cells detected by histopathological assessment through a German board-certified pathologist. A total of 26 patients from our hospital were enrolled, and the baseline clinical information of these patients is shown in Table 1. Ethical approval to conduct this study was granted by the ethics committee of the medical faculty of Magdeburg (33/01, amendment 43/14). Next-generation sequencing was conducted in contract-based cooperation at the genome analytics lab at Helmholtz-Center for Infection Research (HZI) Brunswick, Germany. The tissue miRNA sequence of our lab was defined as OLMS-T, while the serum miRNA sequence of our lab was defined as OLMS-S. The sequenced offline data were subjected to quality control to filter low-quality data while high-quality miRNAs were quantified. This process was completed via miRDeep 2.0.1.2. The workflow is shown in Fig. 1.

**Table1:** Baseline characteristics of enrolled patients.

Characteristics^a^	Overall (*n* = 26)	
	Number	%
Age(years)		
≤65	7	27
>65	19	73
Sex		
Female	16	62
Male	10	38
BMI (Kg/m^**2**^**)**^b^		
≤25	8	31
>25	18	69
Neoadjuvant therapy		
No	15	28
Yes	11	72
Tumor size(mm)		
≤40	10	38
>40	16	62
Surgery		
PPPD	20	77
Others	6	23
Histological type		
PDAC	24	92
IPMN^c^	1	4
NET^d^	1	4
Grade		
1	4	15
2	9	35
3	13	50
Operation complications		
No	16	62
Yes	10	38

^a^All enrolled patients were PDAC.

^b^BMI body mass index.

^c^IPMN intraductal papillary mucinous neoplasm.

^d^NET pancreatic neuroendocrine tumor.

### Public data retrieval and total data pre-processing

A total of eleven datasets are enrolled in our study, including one patient-matching tissue miRNA sequencing dataset of our own laboratory miRNA sequencing tumor dataset (OLMS-T, 13 PC vs. 13 HT samples) and The Cancer Genome Atlas Program-Pancreatic Adenocarcinoma dataset (TCGA-PAAD) (177 PC samples), and nine serum miRNA sequencing datasets, including OLMS-S (13 PC samples), GSE112264 (50 PC vs. 41 HT samples), GSE109319 (24 PC vs. 21 HT samples), GSE113486 (40 PC vs.100 HT samples), GSE59856 (100 PC vs. 150 HT samples), GSE106817 (2759 PC vs. 115 HT samples), GSE85589 (88 PC samples), GSE128508 (10 pancreatitis samples) and GSE128425 (4 pancreatitis samples). For our setup, we defined OLMS-T, GSE112264, and GSE109319 as group one to identify differentially expressed miRNAs between tumors and non-tumor tissues and set GSE113486 and GSE59856 as group two to train the predicted model and test the model robustness. OLMS-S and GSE106817 were used to test the predicted model in independent real-world studies. For group four, the GSE128508, GSE128425 and GSE85589 datasets were used to test the model to identify pancreatitis and PC. The TCGA-PAAD dataset, as group five, was used to explore the clinical value of miRNAs and to predict target genes and potential molecular functions. In addition, because the above data were retrieved from different platforms using different methods, we used the R package sva for batch effect removal in each group. The effect of batch effect removal is presented in Suppl. Fig S1.

### Analysis of differentially expressed miRNAs (DEMs)

We used the limma package to identify differentially expressed miRNAs between normal tissue and cancer tissue from our laboratory sequence data (OLMS-T), healthy serum, and patient serum (GSE112264 and GSE109319). The criteria were set as |Log Fold Change | å 1 and *p* value < 0.01.

### Candidate DEM screening and validation computation in OLM-S sequences

We put three DEM lists into robust rank aggregation (RRA) algorithms to order the importance of DEMs. Variables with a final p value of less than 0.05 were identified as significant DEMs. Considering that these DEMs were enriched according to public datasets, we used our serum dataset (OLMS-S) to validate the expression status of selected miRNAs.

### Machine learning algorithms to build a prediction model using candidate DEMs

We aimed to validate whether the DEMs can identify pancreatic tumors from normal pancreatic tissues. First, according to the 70% vs. 30% ratio, we split the merged datasets from GSE113486 and GSE59856 into a training set and a test set, respectively. Then, four machine learning algorithms, random forest (RF), classification regression tree (CART), support vector machine (SVM), and logistic regression (LR), were used to build the PC risk prediction model in the training set. To test the model robustness, we also combined our laboratory serum (OLMS-S) with public data (GSE106817), and then a new dataset was obtained. Based on this new dataset, we randomly select 50% of the samples to simulate a real-world study to test the model accuracy.

### Testing the pancreatic cancer risk prediction model to discriminate pancreatitis

To compare pancreatitis serum samples (GSE128425 and GSE128508) and GSE85589 containing PC serum samples, bias reduction was implemented to control for differences in clinical and quantity weighing between the three datasets (GSE128425 and GSE85589 using the same platform). Setting GSE128425 and GSE128508 as the control group and GSE85589 as the treatment group, we used propensity score matching (PSM) to control mixed variables between groups and selected 14 samples from the treatment group, which had a comparable age and sex distribution to the control group. After the enrollment of the samples, we validated our model to predict the accuracy between pancreatitis and PC in this new dataset by random forest algorithms.

### Exploring the clinical value of hsa-miR-205-5p

Although the relevance of miR-205 for PC development has been revealed, the work is mostly focused on experimental model systems or ignores to analyze any age, ethnicity, or gender effects on its clinical prognostic value. Moreover, microRNA-205 has been described to be useful as a liquid biopsy diagnostic marker for PC. However, to the best of our knowledge, no report on testing the utility of miR-205 transcript levels as a blood biomarker for PC has been published. To fill these gaps in knowledge, we focused on this marker from the model for further analysis. We used TCGA dataset to validate the clinical value of hsa-miR-205 in tumor samples. Baseline characteristic variables such as sex, age, and race were selected for research to validate hsa-miR-205 expression in different groups. In addition, we also analyzed the association of hsa-miR-205 serum levels with clinical factors, including tumor stage, OS, disease-specific survival (DSS), and progression-free interval (PFI).

### Predicting relevant hsa-miR-205-5p target genes and upstream regulators in our patient cohort data

We used starBase (http://starbase.sysu.edu.cn/), an online tool, to predict candidate targets of hsa-miR-205-5p, followed by gene ontology (GO) enrichment and Kyoto Encyclopedia of Genes and Genomes (KEGG) pathway enrichment analysis (conducted by same name R packages) to explore the molecular mechanism of this miRNA. Applying a strict condition for further data processing (clip dataset to level 3, degradome with level 3, expression in six cancer types, and validation by three programs) limited the computed number of putative candidate genes. We used the Pearson method to analyze the correlation between hsa-mir-205-5p and predicted candidate targets to identify a bona fide key target. The online tool KOBAS (KEGG Orthology-Based Annotation System; http://kobas.cbi.pku.edu.cn/genelist/) was used to conduct the pathway enrichment analysis of the chosen target genes based on the KEGG and Reactome databases.

## Results

### Upregulated miRNAs in pancreatic cancer serum and tissue samples compared to adjacent non-tumor tissues

**Fig. 2:**
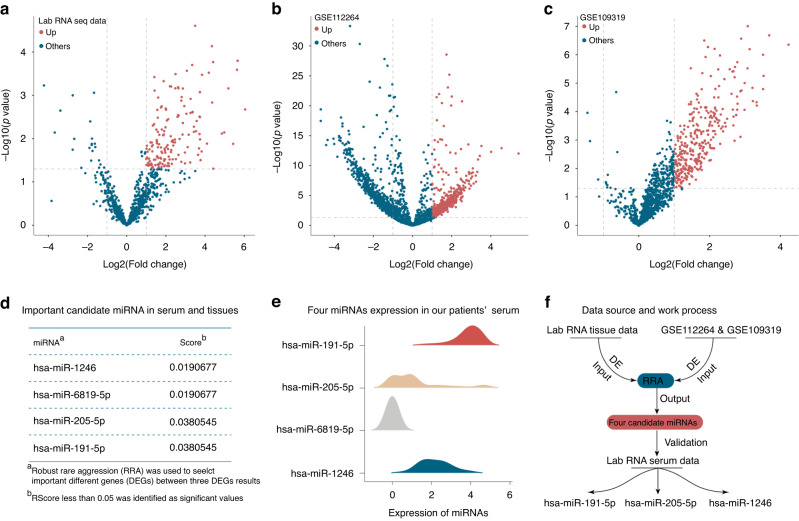
Differential gene expression analysis and feature miRNA identification. Analysis of upregulated miRNAs in our lab data (**a**) and public datasets (**b**, **c**). No merged results of upregulated miRNAs (**d**). RRA algorithms identified a four-feature miRNA panel (**e**), which after validation in our lab dataset was reduced to a three-feature miRNA panel (**f**).

We analyzed a panel of miRNAs between PC normal and tumor tissue in our lab tissue dataset (OLMS-T) and serum datasets from GSE112264 and GSE109319, respectively. The results showed 66 upregulated DEMs in PC tissue in the OLMS-T dataset and 418 and 267 upregulated DEMs in PC serum in the GSE112264 and GSE109319 datasets, respectively (Fig. 2a–c). Merging the above results showed no intersection of miRNAs in either tissue or serum samples. Then, we chose the RRA method to select key miRNAs that showed elevated expression in both tumor tissue and serum. We identified four miRNAs for further processing, including hsa-mir-6819-5p, hsa-mir-1246, hsa-mir-205-5p, and hsa-mir-191-5p (Fig. 2d). However, when we validated the expression of these four miRNAs in patient serum from our own lab, we found that hsa-mir-6819-5p was not detectable and therefore excluded it from the following analysis (Fig. 2e). The work process is depicted in Fig. 2f.

### Serum miRNAs can predict pancreatic cancer presence with high accuracy

**Fig. 3:**
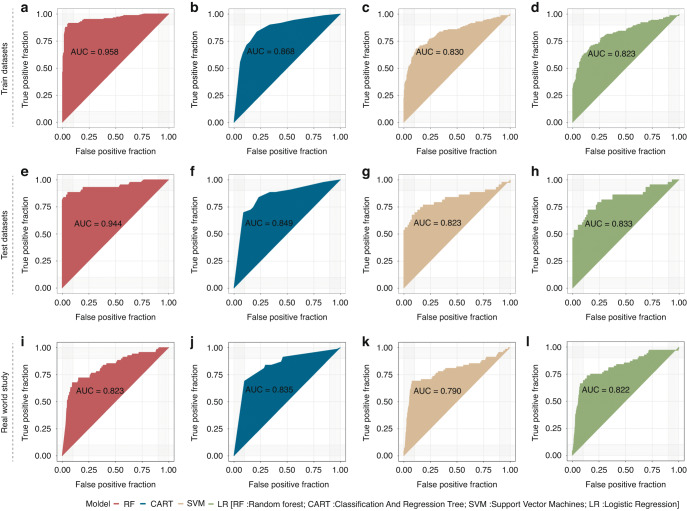
Four machine-learning algorithms validate the predictive efficacy of microRNA panel. Application of the three-miRNA panel to predict pancreatic cancer occurrence in the serum of patients via four machine-learning algorithms (RF, CART, SVM and LR) in the training datasets (**a**–**d**), test datasets (**e**–**h**) and real-world study (**i**–**l**).

In the training set, the RF algorithm showed that three miRNAs (hsa-mir-1246, hsa-mir-205-5p, and hsa-mir-191-5p) could predict PC with a high accuracy of 95.8% (Fig. 3a). CART, SVM, and LR algorithms also showed a high prediction accuracy. The area under the curve values were 0.868, 0.830, and 0.823, respectively (Fig. 3b–d). In the test dataset, these three miRNAs also showed a better prediction accuracy for PC than traditional serum markers. The accuracy of our prediction model of the four methods is 94.4%, 84.9%, 82.3%, and 83.3%, respectively (Fig. 3e–h). In a real-world study, this miRNA signature could distinguish PC patients from healthy patients based on serum samples with an accuracy of 82.3% according to the random forest algorithm (Fig. 3i); the accuracy was 83.5% with CART, 79.0% with SVM, and 82.2% with LR (Fig. 3j–l).

### Serum miRNA signature discriminates pancreatic cancer from chronic pancreatitis

**Fig. 4:**
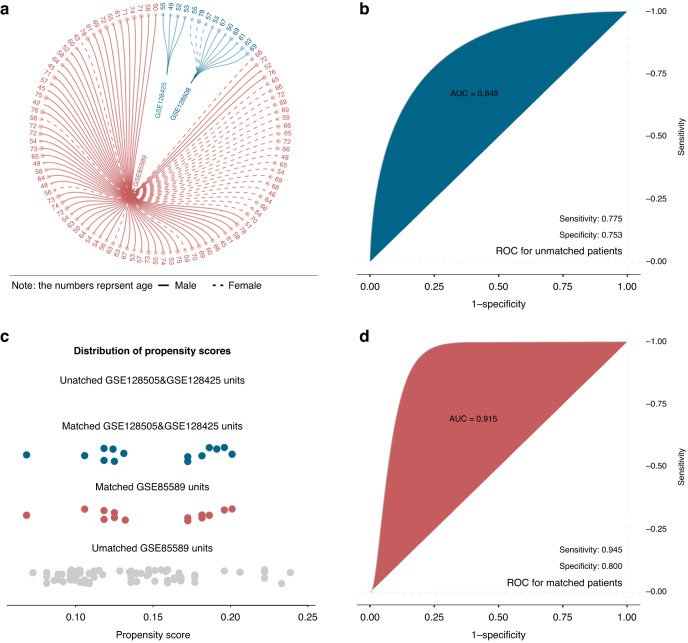
The panel of three serum miRNAs can distinguish pancreatitis from pancreatic cancer. Baseline clinical information of the enrolled patient groups (**a**). The feature miRNA panel prediction ability of 84.5% (**b**), which eliminates baseline differences between groups using propensity score matching (**c**), is increased to very indicative (91.5%, with a sensitivity of 94.5% and specificity of 80.0%) (**d**).

A total of three PC and chronic pancreatitis datasets were enrolled in this study, and Fig. 4a shows the baseline information of these participants. Before PSM, the predictive accuracy of the serum miRNA signature for discriminating between PC and pancreatitis was 84.5% (Fig. 4b). After the propensity score matching study (PSM), all pancreatitis patients (*n* = 14) from the GSE128425 and GSE128508 datasets were successfully harmonized in terms of the baseline clinical characteristics of PC patients (*n* = 14) in the GSE85589 dataset (Fig. 4c). After harmonization, using random forest analysis, the predictability of the miRNA triplet to discriminate pancreatitis from PC in patient serum increased to an accuracy of 91.5% (Fig. 4d).

### High expression of serum hsa-miR-205-5p is associated with a poor clinical outcome and incomplete tumor resection

**Fig. 5:**
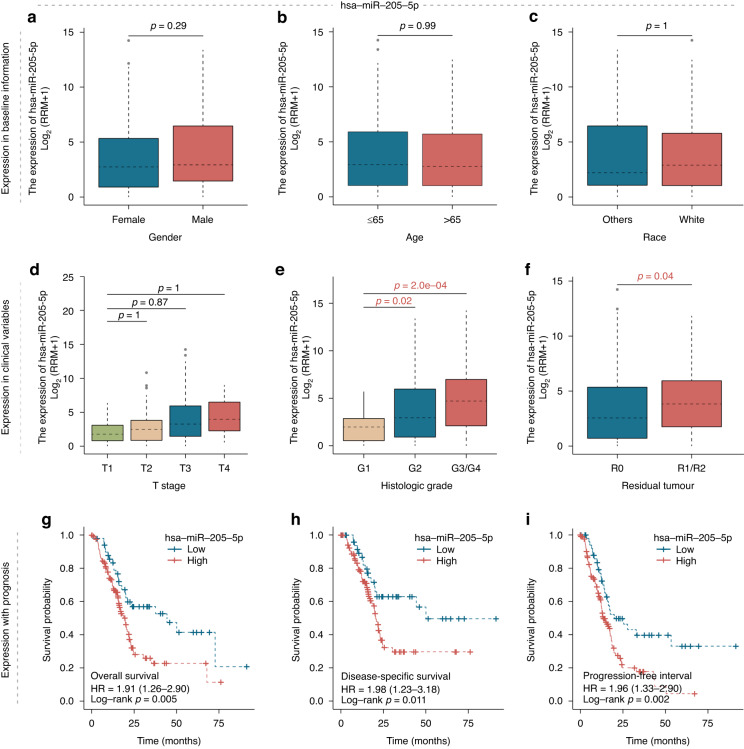
Association of the novel serum biomarker hsa-miR-205 with clinical factors. Subgroup analysis to determine whether the hsa-miR-205 expression difference is independent of other factors: subgroups based on sex, age and race of the patient (**a**–**c**), subgroups based on T stage, histologic grade, and residual tumor status (**d**–**f**). Survival analysis of serum hsa-miR-205-5p expression level as an independent prognostic factor revealed that high expression is associated with significantly poorer clinical outcomes, namely, reduced OS, DSS or PFI (**g**–**i**).

When we analyzed hsa-miR-205-5p expression in the TCGA-PAAD cohorts, we found that there was no significant difference (Fig. 5a–c) among sex, age, or race. Although there was no statistical significance, we detected an increase in transcript abundance in patients with elevated T stage or advanced histologic grading stage of the disease (Fig. 5d, e). The R0 resection margin of the tumor specimen is considered an envisioned outcome of curative-intent oncological surgery. We found that serum hsa-miR-205-5p expression was higher in those with R1/2 resection than in those with R0 resection (Fig. 5f). These results demonstrate that hsa-miR-205-5p could be used as a predictive marker for more advanced disease. Moreover, in the survival analysis, patients with high hsa-miR-205-5p expression always had a worse prognosis than patients with low hsa-miR-205-5p expression in terms of OS, DSS, and PFI (*p* = 0.05, *p* = 0.011, and *p* = 0.002, respectively) (Fig. 5g–i).

### Predicting upstream and downstream targets of hsa-miR-205-5p in pancreatic cancer

**Fig. 6:**
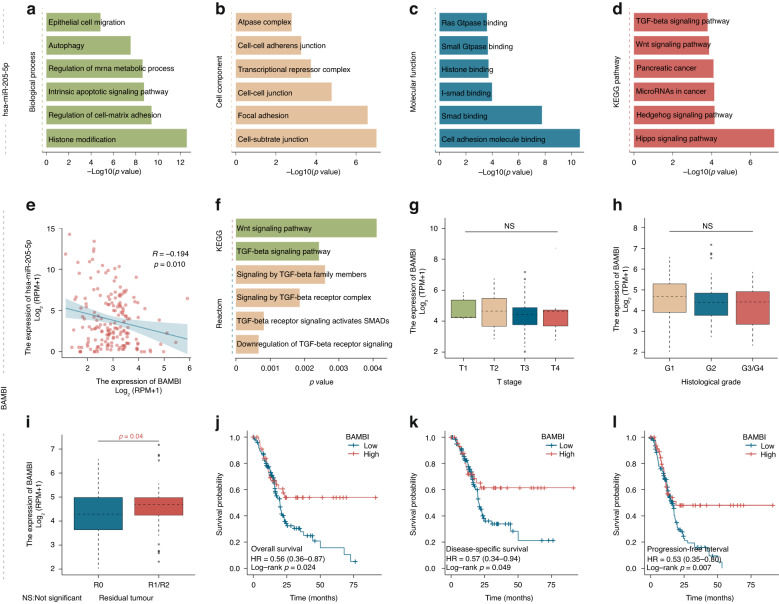
Prediction of the mechanistic function of hsa-miR-205 and its candidate target BAMBI by interrogating mRNA and small RNA datasets. The results of gene ontology analyses and pathway enrichment analyses for hsa-miR-205-related genes (**a**–**d**), which led to the identification BAMBI transcription as a putative target of hsa-miR-205 (**e**). Prediction of biological pathways involving BAMBI transcription in pancreatic cancer (**f**), as well as the distribution of tumor grading features including T stage (**g**), histologic grade (**h**) and residual tumor stage (**i**) in patients with different values of BAMBI transcription levels. Survival analysis of colon cancer patients with different BAMBI mRNA levels in their tumors, revealing that high BAMBI expression was associated with better clinical outcomes in terms of OS, DSS and PFI (**j**–**l**).

After limiting the stringency of CLIP (crosslinking-immunoprecipitation) data to more than three, a total of 1616 candidate targets of hsa-miR-205-5p were predicted (Supplementary Table 1), and gene ontology enrichment analysis showed that these targets could be enriched in epithelial cell migration, autophagy and cell‒cell adherens junctions (Fig. 6a–c). Future study of putative signaling pathway analysis indicates that the above targets may be involved in the TGF-beta signaling pathway as well as phylogenetically conserved stem cell signaling pathways such as Wnt and Hedgehog signaling (Fig. 6d).

To identify putative downstream targets, a total of 13 target genes were enrolled. Strikingly, after correlation analysis between hsa-miR-205-5p and its targets in PC, we found that only the expression of BMP and Activin Membrane-Bound Inhibitor (BAMBI) was significantly (negatively) associated with the expression level of hsa-miR-205-5p (Supplementary Figs. 2 and 6e). Therefore, BAMBI was identified as a candidate target for further analysis. When we explored the potential mechanism of the BAMBI gene in PC, the pathway enrichment results indicated that this gene may be involved in the Wnt signaling pathway and TGF-beta signaling pathway (Fig. 6f). Considering that hsa-miR-205-5p is also enriched in the same pathways, we could infer that hsa-miR-205-5p may target BAMBI to activate the TGF-beta pathway, which in turn promotes pancreatic carcinogenesis.

### High mRNA expression of BAMBI is associated with better clinical outcomes for patients with pancreatic cancer

Since BAMBI might be regulated by hsa-miR-205-5p, its expression trend should be negatively correlated with the expression of hsa-miRNA 205-5p. To demonstrate this hypothesis, we used the TCGA-PAAD database to conduct relevant data assessment. In concordance, we found that as the disease stage increased, the expression level of this gene decreased (Fig. 6g–i). Moreover, survival analysis results showed that high mRNA expression of BAMBI in PC indicated a better outcome, including prolonged OS, DSS and PFI (*p* = 0.024, *p* = 0.049, and *p* = 0.007, respectively) (Fig. 6j–l).

## Discussion

Early detection of disease manifestation or progression is a clinically feasible type of prevention scheme for malignant diseases. Current science policymakers show an increased focus on funding programs to develop better disease detection modalities [20]. A large amount of evidence from clinical studies indicates the power of early cancer detection based on quantifying CTCs or ctDNAs in liquid biopsies. However, for PC, those approaches seem inefficient or at least infrequently reported because of their extreme rarity in the blood and the difficulty in detection, enumeration and characterization [21]. Currently, the detection rate of ctDNA in early stage (stage I and II) PC does not exceed 30% [22]. Moreover, it is difficult to detect ctDNA due to the low concentration of ctDNA in the blood of patients in early staged of PC. Therefore, the quantification of ctDNA for the early diagnosis of PC is still challenging [23]. In recent years, the early diagnostic value of exosomal miRNA has also received attention, but the difficulty of exosome isolation and purification hinders its further promotion at this point. In contrast, assessing circulating miRNAs does not have the above limitations and can be fundamental for the development of applicable assays with high detection sensitivity and specificity. Our work associates with this field of research using patient-matched multisample collection and thereby supports non-targeted RNA biomarker discovery.

Based on the evidence of the association between miRNAs and various cancers, in recent years many miRNAs present in body fluids (e.g., plasma, cerebrospinal fluid, saliva, urine, semen) have been proposed as potential cancer biomarkers for diagnosis and prognosis [24]. Of note, our goal was to seek diagnostic miRNAs that can specifically address the unmet needs in the clinical diagnosis of PC patients, namely, the difficulty in discriminating tumors from pancreatitis and sensitively detecting the degree of resection of the tumor. The main result of our work is the development of a three-miRNA (hsa-miR-1246, hsa-miR-205-5p, and hsa-miR-191-5p) signature that addresses these issues. The generation and validation in multicenter, large-scale datasets was conducted by unsupervised computational methods (four machine learning algorithms, namely, RF, CART, SVM and LR). Our selective approach contrasts with that of other previously published works on related matters, and we hypothesize that it is of higher clinical translational relevance. Previous studies aimed at developing miRNA-based PC diagnostics are technologically limited, as they did not integrate the real-world datasets [25–27] or they relied on somewhat outdated computational algorithms [28–32]. Moreover, others have suggested a circulating three microRNA panel (miR-642b, miR-885-5p, and miR-22) for blood-based detection of PC [33]. Our sequencing analysis could not confirm their candidate biomarkers. Although it is well known that the biological characteristics of cancers are complex and cannot be attributed to a single factor, recent statistical power calculations across multiple translational PC studies suggest that a single circulating microRNA can hold significant clinical predictive value [34]. In this regard, the hsa-miRNA 205-5p proposed here has previously been reported to regulate PC cell biology, surprisingly with mostly tumor suppressive functions [35, 36], but also pro-tumorigenic functions described [37]. We extend the evidence on this marker, supporting its potential as a new approach for the early diagnostics of PC.

To the best of our knowledge, another novel aspect of our work is the discovery of transcriptional activation of BAMBI as a potential target for PC, and this could be a consequence of epigenetic dysregulation related to the disease. BAMBI was identified as a repressor gene of TGF-beta signaling and was regulated by hsa-miR-205-5p. Since the TGF-beta signaling pathway plays fundamental roles in tumor progression [38], our work reveals new opportunities to investigate the biological function of hsa-miR-205-5p activity in PC. Of note, a study [39] from a decade ago proposed BAMBI to promote pancreatic tumor metastasis in TGF-intact tumors; however, there is no report of its mechanism. Wet lab experiments focusing on deciphering the biological role of transcriptional activation of BAMBI in PC are ongoing and might help to verify our hypothesis regarding this novel therapeutic target in the near future.

We acknowledge the limitations of our study: 1. Our discovery cohort has a relatively low number of patients enrolled from a single center, and the clinical outcome data rely on public datasets only. To address this issue, a dedicated prospective clinical trial benchmarking the value of the three candidate microRNAs in PC patients with clinical follow-up is needed. The design of this trial might include a subgroup analysis discriminating between peri-operative chemotherapy of the patient or different surgery types that might be applied, i.e., robotic vs. laparoscopic. In this context, the most relevant clinical development would be the development of a minimally invasive, sensitive, and specific detection tool to identify pancreatic intraepithelial neoplasia or intraductal papillary mucinous neoplasms to detect PC development in the early stage. A possible trial shall include the option to enroll those patients and analyze their blood, as the disease is currently mostly identified by coincidental diagnosis. Our conducted literature search did not identify any microRNA dataset for this disease in the public domain that could be interrogated in our study. Moreover, due to insufficient depth of available data on the DNA mutation status of our analyzed tumor samples, we cannot confirm any utility of our test to discriminate among the genetic tumor subtypes. 2. Biomarker analysis is based on ultrasensitive next-generation sequencing data acquisition. The execution of a wet lab confirmatory trial, ideally on prospectively collected samples as described above, using targeted RNA quantification, such as RT‒qPCR or *CRISPR/Cas* diagnostics, is useful to validate our discovery [40].

## Conclusions

In this study, a panel of three miRNAs (hsa-miR-1246, hsa-miR-205-5p, and hsa-miR-191-5p) was used to predict PC independent of the disease stage. Quantifying the transcript levels of hsa-miRNA 205-5p in the serum of patients might help to improve the stratification of tumor patients from patients with pancreatitis and can help to inform surgeons of the completeness of resection of the tumor area in a perioperative setting. The underlying mechanism and the utility of the proposed strategy to identify tumor subtypes need to be evaluated in follow-up studies.

### Supplementary information

Supplement Figure 1

Supplement Figure 2

Supplementary File Legends

Supplement Table 1

## Data Availability

All data can be obtained from the corresponding author.

## Code availability

All code with analysis can be obtained from the corresponding author.
