# Supplementary figures and images for: Integrating a microRNA signature as a liquid biopsy-based tool for the early diagnosis and prediction of potential therapeutic targets in pancreatic cancer
Source: Br J Cancer. 2023 Nov 10;130(1):125–34. doi: 10.1038/s41416-023-02488-4 (PMC10781694; doi:10.1038/s41416-023-02488-4)

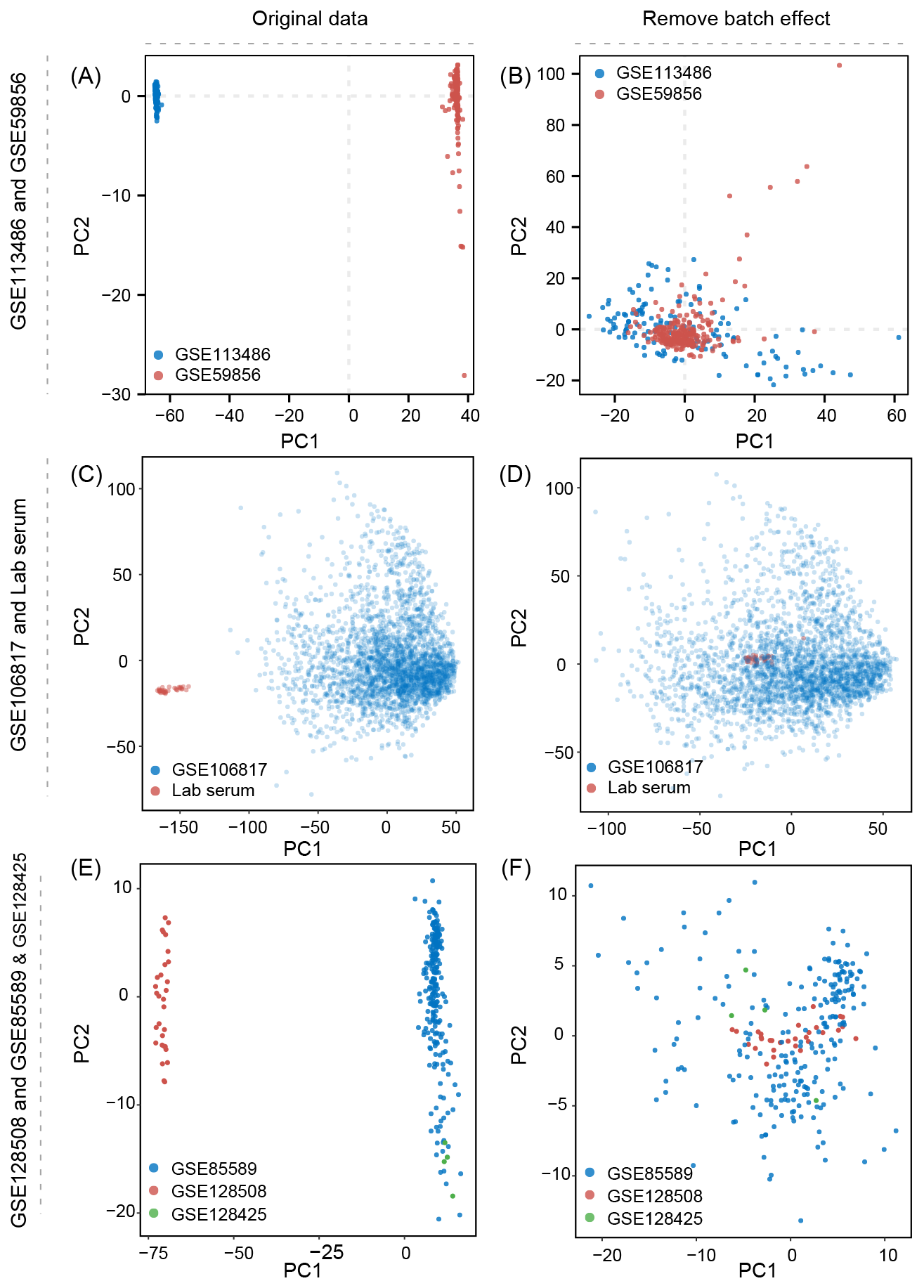

Supplement: Supplementary file 1 — Supplement Figure 1 [file 41416_2023_2488_MOESM1_ESM.tif]

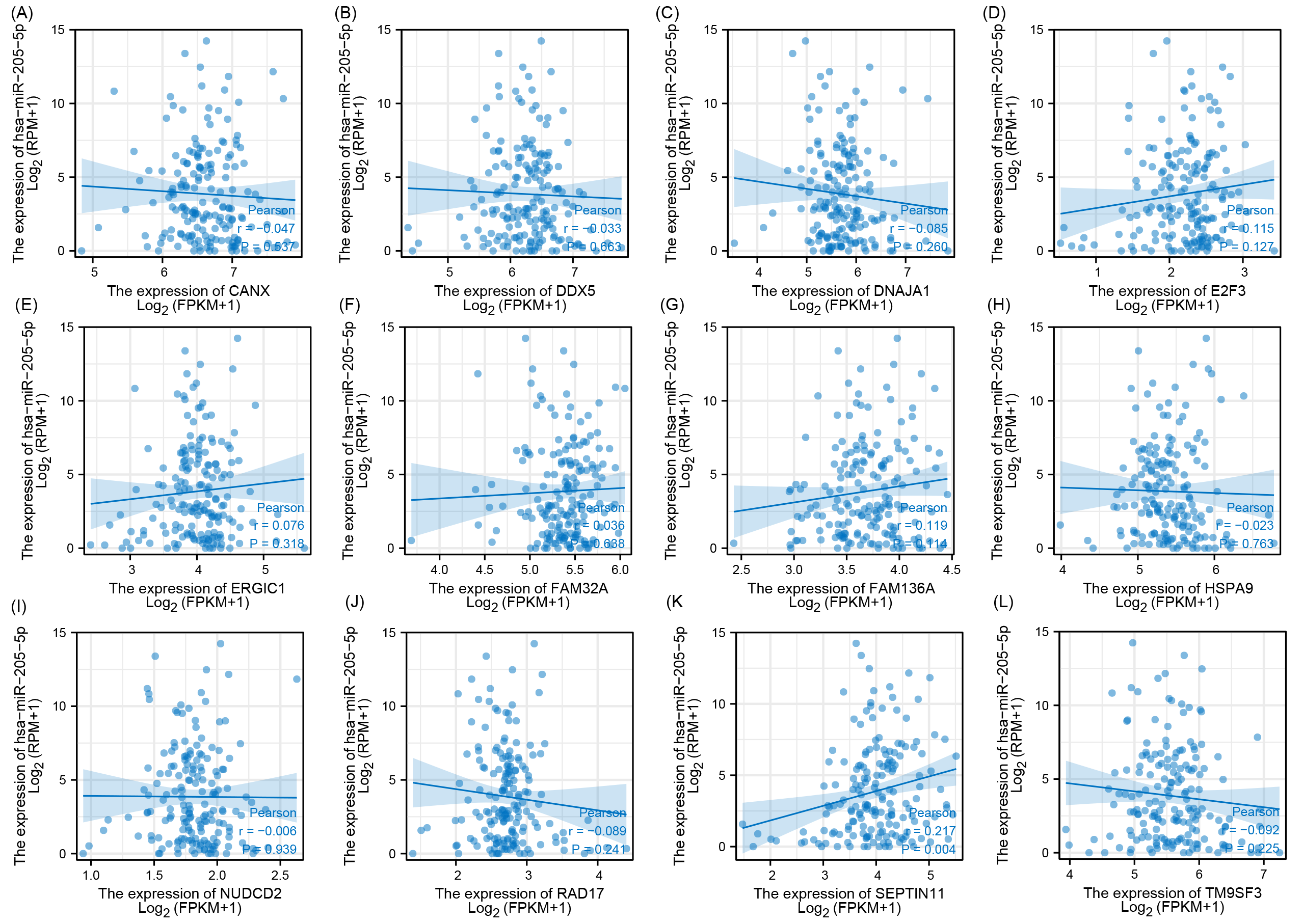

Supplement: Supplementary file 2 — Supplement Figure 2 [file 41416_2023_2488_MOESM2_ESM.tif]
