# Supplementary material for: Integrating a microRNA signature as a liquid biopsy-based tool for the early diagnosis and prediction of potential therapeutic targets in pancreatic cancer
Source: Br J Cancer. 2023 Nov 10;130(1):125–34. doi: 10.1038/s41416-023-02488-4 (PMC10781694; doi:10.1038/s41416-023-02488-4)
Supplement: Supplementary file 3 — Supplementary File Legends [file 41416_2023_2488_MOESM3_ESM.docx]

**Figure S1** Signal distribution before and after removing batch effects between the GSE113486 and GSE59856 datasets (A&B), before and after removing batch effects between GSE106817 and our lab serum datasets (C&D), and before and after removing batch effects between the GSE128425, GSE128508 and GSE85589 datasets (E&F).

**Figure S2** Correlation coefficient between hsa-miR-205 expression and the expression of selected candidate targets.
