# Supplementary material for: Integrating a microRNA signature as a liquid biopsy-based tool for the early diagnosis and prediction of potential therapeutic targets in pancreatic cancer
Source: Br J Cancer. 2023 Nov 10;130(1):125–34. doi: 10.1038/s41416-023-02488-4 (PMC10781694; doi:10.1038/s41416-023-02488-4)
Supplement: Supplementary file 4 — Supplement Table 1 [file 41416_2023_2488_MOESM4_ESM.docx]

| **Supplement Table 1 A total of 1616 candidate targets of hsa-miR-205-5p** | |
| --- | --- |
| **miRNA** | hsa-miR-205-5p |
| **Targets** | DDX3Y,RPL22,KLHL21,ERRFI1,USP48,ZNF436,WASF2,PUM1,AK2,PHC2,MYCBP,FOXJ3,HECTD3,PIK3R3,EPS15,C1orf123,DHCR24,TACSTD2,DDAH1,TMED5,SLC25A24,RHOC,TRIM33,BCAS2,NRAS,CSDE1,SIKE1,GDAP2,NOTCH2,PRKAB2,ANP32E,GATAD2B,DENND4B,CRTC2,SLC39A1,ADAR,PBXIP1,PYGO2,FAM189B,KHDC4,SSR2,SMG5,GPATCH4,MRPL24,HDGF,DCAF8,SLC19A2,TOR1AIP2,STX6,IVNS1ABP,TPR,PHLDA3,PPP1R15B,DSTYK,ELK4,NUCKS1,YOD1,LPGAT1,TP53BP2,LBR,ENAH,PYCR2,TRIM11,CCSAP,NID1,GREM2,CHML,CEP170,HNRNPU,AHCTF1,SAMD11,KIF1B,MFN2,PRDM2,KAZN,DNAJC16,SZRD1,OTUD3,ZBTB40,PNRC2,CLIC4,SH3BGRL3,ARID1A,ZDHHC18,PPP1R8,EPB41,KPNA6,TXLNA,AGO1,DMAP1,NASP,RNF11,BTF3L4,ZYG11B,NFIA,LEPR,MIER1,CYR61,LMO4,FNBP1L,ABCD3,SLC30A7,PRMT6,GNAI3,CSF1,STRIP1,CAPZA1,PTGFRN,MAN1A2,FAM46C,AC239799.1,NOTCH2NL,SNX27,ZBTB7B,ADAM15,EFNA1,LMNA,DUSP23,UHMK1,UAP1,PBX1,POGK,POU2F1,SUCO,TOR3A,SOAT1,TOR1AIP1,CEP350,XPR1,LAMC1,RGS2,NEK7,CAMSAP2,NAV1,TIMM17A,ATP2B4,ZC3H11A,ZBED6,MDM4,IL24,CD55,DTL,CENPF,RRP15,SRP9,ARF1,IBA57,ARV1,MTR,ZBTB18,ASB13,UPF2,YME1L1,ACBD5,ARHGAP12,ZNF248,CXCL12,SGMS1,JMJD1C,HERC4,DNAJC9,WAPL,PANK1,CPEB3,TM9SF3,MMS19,GOT1,SH3PXD2A,GPAM,GFRA1,TIAL1,MCMBP,MKI67,PFKFB3,GATA3,DHTKD1,CDC123,VIM,BAMBI,ZEB1,CCNY,BICC1,HNRNPH3,TET1,CCAR1,HK1,EIF4EBP2,SGPL1,DDIT4,MCU,VCL,PPIF,PTEN,KIF20B,TBC1D12,HELLS,CCNJ,ZNF518A,LCOR,CUTC,SCD,HIF1AN,SLK,DUSP5,NHLRC2,FAM160B1,BCCIP,INPP5A,TOLLIP,NAP1L4,SBF2,HPS5,FANCF,CD59,AL049629.2,ARHGAP1,CKAP5,CELF1,TNKS1BP1,PRPF19,DDB1,CPSF7,GANAB,ATG2A,CDCA5,SYVN1,C11orf68,FAM168A,PGM2L1,CREBZF,SYTL2,PICALM,TMEM123,MMP3,MSANTD4,KDELC2,USP28,CADM1,IFT46,DDX6,HYOU1,H2AFX,NECTIN1,HSPA8,CDON,SRPRA,ILK,EIF3F,TUB,IPO7,CTR9,USP47,TEAD1,PDE3B,NAV2,QSER1,HIPK3,CAT,PDHX,CD44,FJX1,TRIM44,LDLRAD3,SLC35C1,PTPRJ,CTNND1,ZFP91,TTC9C,RTN3,RPS6KA4,CDC42EP2,PELI3,CCND1,CTTN,RNF121,INPPL1,PRSS23,JRKL,YAP1,CUL5,ZC3H12C,DLAT,PAFAH1B2,UBE4A,KMT2A,ARCN1,HMBS,CBL,OAF,ARHGEF12,TBCEL,EI24,APLP2,KDM5A,VAMP1,MLF2,LRP6,KCNJ8,BCAT1,ERGIC2,TMTC1,CAPRIN2,YARS2,GXYLT1,PUS7L,SLC38A1,SLC38A2,ASB8,CCNT1,TUBA1B,FMNL3,SLC11A2,SMAGP,KRT80,ITGA5,RNF41,ANKRD52,BAZ2A,ZBTB39,DCTN2,LRIG3,PPM1H,C12orf66,NAP1L1,UBE2N,TMCC3,CDK17,NT5DC3,SART3,GIT2,ANAPC7,MED13L,PXN,TRIAP1,VPS33A,AC048338.1,CLIP1,VPS37B,MPHOSPH9,CDK2AP1,SCARB1,GOLGA3,ERC1,FKBP4,CCND2,TIGAR,PTMS,NECAP1,APOLD1,DDX47,H2AFJ,STRAP,AEBP2,ETNK1,MED21,STK38L,DNM1L,ANO6,ARID2,SMARCD1,DIP2B,ACVR1B,SP1,HNRNPA1,ERBB3,LRP1,9-Mar,SRGAP1,RASSF3,HMGA2,CAND1,NUP107,CPSF6,FRS2,CCT2,C12orf29,MRPL42,DRAM1,TXNRD1,FICD,UNG,ACACB,SH2B3,ERP29,PTPN11,OAS2,SUDS3,MLEC,RNF34,BCL7A,MLXIP,DDX55,RAN,XPO4,SACS,MTMR6,SLC7A1,KATNAL1,N4BP2L2,STARD13,ELF1,KBTBD7,TSC22D1,RCBTB1,SPRYD7,INTS6,KCTD12,MYCBP2,DOCK9,COL4A1,PCID2,MRPL57,NUP58,RPL21,GTF3A,NHLRC3,AKAP11,FNDC3A,UTP14C,BORA,MBNL2,IPO5,TM9SF2,ZIC2,COL4A2,ATP11A,LAMP1,RBM23,STRN3,CFL2,KLHL28,MIS18BP1,L2HGDH,NIN,ERO1A,FERMT2,DDHD1,CNIH1,RAB15,MAX,VTI1B,RDH11,ZFYVE26,DCAF5,SYNJ2BP,MAP3K9,ELMSAN1,AREL1,IRF2BPL,VIPAS39,SPTLC2,SNW1,SEL1L,PTPN21,RPS6KA5,MOAP1,DICER1,ATG2B,HSP90AA1,CDC42BPB,CKB,ABHD4,MMP14,BCL2L2,KHNYN,FAM177A1,GEMIN2,PNN,ARF6,SAMD4A,MAPK1IP1L,ARID4A,DAAM1,PCNX4,PLEKHH1,SUSD6,SRSF5,PCNX1,SIPA1L1,RBM25,ZNF410,AC005520.1,GPATCH2L,CIPC,CALM1,CPSF2,CCNK,PPP2R5C,MTA1,NIPA2,MTMR10,BMF,ZNF106,UBR1,MFAP1,SECISBP2L,DMXL2,MYO5C,MYO5A,CCPG1,PRTG,RFX7,ADAM10,SLTM,RORA,CA12,CSNK1G1,RBPMS2,INTS14,LARP6,ADPGK,SEMA7A,PEAK1,BTBD1,POLG,HDDC3,FAM174B,THBS1,MGA,SERF2,CASC4,CTDSPL2,DUT,EID1,MINDY2,RAB8B,ZNF609,SMAD3,GLCE,PAQR5,ARID3B,CLK3,SCAMP5,SNX33,FBXO22,HMG20A,PSMA4,TMED3,ABHD17C,CEMIP,ABHD2,ZNF710,NGRN,AC091167.2,IQGAP1,CHD2,NR2F2,IGF1R,PGP,GLYR1,EMP2,SMG1,DCUN1D3,USP31,EARS2,CDIPT,MAPK3,DCTPP1,SIAH1,CSNK2A2,CDH11,CMTM4,DYNC1LI2,ESRP2,CHTF8,PDF,COG8,NQO1,PHLPP2,AP1G1,ZFHX3,RFWD3,TMEM170A,ADAT1,MBTPS1,ZDHHC7,ZCCHC14,ANKRD11,CHMP1A,FANCA,RAB11FIP3,STUB1,JPT2,CCNF,KCTD5,ZNF263,VASN,MGRN1,ERCC4,PDXDC1,TMC7,ITPRIPL2,METTL9,MOSMO,DCTN5,NFATC2IP,PAPD5,ARL2BP,C16orf70,NUTF2,SLC7A6,CDH1,TANGO6,HAS3,SNTB2,VPS4A,NFAT5,WWP2,ATXN1L,IST1,ZFP1,GAN,GSE1,COX4I1,FOXF1,CDT1,CDK10,AC092143.1,TUBB3,YWHAE,ANKFY1,PELP1,DHX33,MYH10,FLCN,USP22,PIGS,NUFIP2,CRLF3,ACACA,DDX52,MED1,CCR7,ACLY,DNAJC7,KAT2A,CAVIN1,DUSP3,CCDC43,MAP3K14,PLEKHM1,CDC27,PHB,PPP1R9B,LRRC59,ANKRD40,MBTD1,COX11,MMD,CUEDC1,VEZF1,MTMR4,TRIM37,SKA2,USP32,APPBP2,MED13,CYB561,ICAM2,DDX5,GNA13,AXIN2,AC004805.1,FAM104A,GGA3,TIMP2,CANT1,CBX4,TBC1D16,EIF4A3,FASN,CSNK1D,PAFAH1B1,ARRB2,MINK1,ZFP3,RABEP1,TTC19,ALDH3A2,TRAF4,TAOK1,CPD,SUZ12,ZNF207,SLFN5,AP2B1,AATF,CISD3,LASP1,MSL1,RARA,NKIRAS2,PSME3,NBR1,NMT1,NSF,KPNB1,NFE2L1,CALCOCO2,UBE2Z,IGF2BP1,KAT7,LUC7L3,MSI2,PPM1E,PRR11,YPEL2,CLTC,BCAS3,METTL2A,DCAF7,MAP3K3,DDX42,PRKCA,BPTF,KCTD2,SEC14L1,9-Sep,TNRC6C,RNF213,YES1,PPP4R1,ABHD3,SS18,DSC2,B4GALT6,ZNF24,RPRD1A,SMAD2,C18orf32,RPL17-C18orf32,MYO5B,BCL2,TMX3,ZADH2,TGIF1,PTPRM,RAB31,TUBB6,SEH1L,CEP192,RNMT,ELP2,SMAD4,AC091551.1,NEDD4L,PMAIP1,SOCS6,MKNK2,LMNB2,DIRAS1,MYDGF,INSR,ELAVL1,LSM4,ISYNA1,CERS1,CEBPA,ZNF566,ZNF607,EID2B,SERTAD3,IRGQ,ZNF45,CARD8,MIDN,CIRBP,LDLR,CALR,NACC1,ZSWIM4,ADGRE5,TPM4,RAB8A,FAM32A,KLF2,KXD1,UPF1,MAU2,GATAD2A,ZNF90,ZNF507,KCTD15,ZNF302,FXYD5,LSR,COX6B1,MRPS12,SNRPA,ZNF574,ZNF234,APOC1,SAE1,FTL,ATF5,EMC10,ZNF444,ZNF264,ZNF460,KIDINS220,PDIA6,ROCK2,SMC6,WDR35,DNMT3A,ASXL2,CYP1B1,THADA,FBXO11,USP34,SERTAD2,PPP3R1,GFPT1,FAM136A,DCTN1,MOGS,CCDC142,MRPL53,EVA1A,TGOLN2,IMMT,KANSL3,SEMA4C,MGAT4A,LONRF2,RGPD6,BUB1,CCDC93,CLASP1,MAP3K2,POLR2D,FAM168B,ZEB2,RND3,BAZ2B,RBMS1,TLK1,NEMP2,STAT1,HECW2,SATB2,TRAK2,ALS2,RAPH1,INO80D,METTL21A,USP37,ATG9A,AC068946.2,PER2,GRHL1,GREB1,NCOA1,CLIP4,YIPF4,GEMIN6,PRKCE,EPAS1,EPCAM,MSH2,MSH6,MXD1,PCYOX1,ZNF638,DOK1,MRPL19,TMSB10,INPP4A,EIF5B,GCC2,RANBP2,SOWAHC,RGPD5,TMEM87B,PSD4,PLEKHB2,CCNT2,UBXN4,SPOPL,ACVR2A,LYPD6,RIF1,FMNL2,GPD2,PKP4,CERS6,PDK1,UBE2E3,SSFA2,BZW1,CFLAR,BMPR2,FAM117B,ABI2,PIKFYVE,CAB39,ARMC9,COPS7B,ATG16L1,MLPH,ASB1,2-Sep,TBC1D20,CSNK2A1,FKBP1A,NSFL1C,C20orf194,SLC23A2,GPCPD1,JAG1,NAPB,BCL2L1,PLAGL2,E2F1,RBM12,RBM39,DSN1,ZHX3,OSER1,SERINC3,SDC4,ZNFX1,B4GALT5,SPATA2,TMEM189-UBE2V1,UBE2V1,ATP9A,SALL4,PMEPA1,PRELID3B,PPP1R3D,ZNF512B,ZCCHC3,NOP56,CDC25B,PRNP,PLCB1,BTBD3,SNRPB2,PCSK2,SMIM26,POFUT1,KIF3B,MAPRE1,ACSS2,EPB41L1,AAR2,TGIF2,RALGAPB,TOP1,PLCG1,TTPAL,NCOA3,PARD6B,CSTF1,RAB22A,GNAS,MRGBP,GID8,TPD52L2,HSPA13,NRIP1,APP,N6AMT1,CCT8,SETD4,VPS26C,WDR4,SUMO3,PTTG1IP,LSS,GABPA,USP16,IFNAR2,IFNAR1,SON,SLC5A3,SIM2,TTC3,DYRK1A,ABCG1,RRP1B,TRAPPC10,FP565260.7,ADARB1,COL18A1,HDHD5,PPM1F,GUCD1,PITPNB,XBP1,NIPSNAP1,PATZ1,RBFOX2,TST,CSNK1E,DDX17,ST13,TOB2,POLDIP3,RTL6,COMT,DGCR8,PPIL2,CCDC117,ZNRF3,MTMR3,YWHAH,HMOX1,APOL6,H1F0,POLR2F,MIEF1,TNRC6B,ADSL,AL022238.4,L3MBTL2,IQSEC1,RBSN,THRB,TOP2B,AZI2,HIGD1A,CDCP1,PRKAR2A,QARS,IP6K1,TUSC2,ABHD14B,DUSP7,DCP1A,ACTR8,FAM208A,DENND6A,LRIG1,FOXP1,TOMM70,ATG3,USF3,NAA50,B4GALT4,GSK3B,LRRC58,FSTL1,GOLGB1,WDR5B,KPNA1,MYLK,ITGB5,ZNF148,SNX4,MGLL,TMCC1,MSL2,STAG1,XRN1,WWTR1,B3GALNT1,PDCD10,MECOM,PHC3,TBL1XR1,MAGEF1,TMEM41A,BCL6,TFRC,RUBCN,BHLHE40,ARL8B,THUMPD3,BRK1,TATDN2,AC022384.1,TMEM43,NR2C2,EAF1,UBE2E1,RPL15,TGFBR2,CMTM7,CRTAP,PDCD6IP,ACVR2B,EXOG,WDR48,RPSA,RPL14,CTNNB1,KLHL18,WDR6,GNAI2,PTPRG,KBTBD8,ARL13B,CMSS1,CEP97,PHLDB2,ABHD10,ATP6V1A,TIMMDC1,UMPS,PLXNA1,SRPRB,ARMC8,ZBTB38,SELENOT,MED12L,RAP2B,TIPARP,PTX3,GFM1,PPM1L,SKIL,CLDN11,USP13,PSMD2,POLR2H,MAP3K13,SENP2,EIF4A2,LPP,FBXO45,PAK2,NCBP2-AS2,FYTTD1,CTBP1,LETM1,LRPAP1,GRPEL1,AFAP1,ZNF518B,HS3ST1,RAB28,TAPT1,SMIM14,PDS5A,RBM47,FRYL,CLOCK,GRSF1,HNRNPD,TMEM150C,SCD5,KLHL8,HSD17B11,PYURF,ADH5,DNAJB14,CAMK2D,ANKRD50,SLC7A11,OTUD4,ZNF827,NR3C2,TMEM154,CASP3,ACSL1,FAT1,FGFRL1,NSD2,RNF4,ADD1,MSX1,MRFAP1,NCAPG,STIM2,UBE2K,PAICS,REST,MOB1B,EREG,SHROOM3,11-Sep,AFF1,HERC3,SMARCAD1,PDLIM5,DAPP1,SGMS2,AP1AR,EXOSC9,KIAA1109,SCOC,USP38,ABCE1,SMAD1,MMAA,MND1,FNIP2,RAPGEF2,KLHL2,GALNT7,LPCAT1,MYO10,GOLPH3,ZFR,RPL37,C5orf34,MOCS2,IL6ST,MIER3,PLK2,TAF9,ENC1,LHFPL2,ARSB,MTX3,EDIL3,TMEM161B,LYSMD3,FAM172A,ELL2,PJA2,EPB41L4A,REEP5,ATG12,HINT1,RAPGEF6,AC008695.1,IRF1,AFF4,ETF1,HSPA9,PCDH1,SPRY4,CSNK1A1,DCTN4,RNF145,NUDCD2,UBTD2,NHP2,CLK4,RNF130,MTRR,CCT5,TRIO,C5orf22,TARS,NIPBL,C5orf51,ZNF131,MRPS30,SNX18,GPX8,MAP3K1,GPBP1,PPWD1,AC008560.1,NLN,ERBIN,PIK3R1,SLC30A5,RAD17,MAP1B,TNPO1,HMGCR,POLK,ZFYVE16,RGMB,PAM,APC,DCP2,COMMD10,HSD17B4,ISOC1,RAD50,AC116366.3,KDM3B,CTNNA1,PAIP2,ADRB2,GRPEL2,SLC26A2,GPX3,G3BP1,GALNT10,LARP1,MRPL22,WWC1,RARS,SPDL1,ERGIC1,CREBRF,SFXN1,ARL10,FAF2,TMED9,CANX,MAML1,SQSTM1,CNOT6,TUBB2A,SSR1,DEK,DCDC2,DDX39B,ATP6V1G2-DDX39B,CLIC1,NEU1,LEMD2,TAF11,TEAD3,SRPK1,STK38,CCND3,XPO5,MCM3,TRAM2,DST,RAB23,EEF1A1,COL12A1,TMEM30A,FAM46A,IBTK,AKIRIN2,RNGTT,LYRM2,MAP3K7,MMS22L,CCNC,ATG5,CDK19,REV3L,TSPYL4,TSPYL1,CEP85L,MAN1A1,ECHDC1,ARHGAP18,NHSL1,LATS1,LRP11,MTRF1L,EZR,MPC1,PHF10,FOXC1,PRPF4B,RREB1,SMIM13,TMEM170B,CD83,JARID2,E2F3,SOX4,ZKSCAN8,HLA-A,ABCF1,BRD2,ANKS1A,CDKN1A,C6orf89,RNF8,VEGFA,HSP90AB1,ENPP4,PTP4A1,PHF3,SMAP1,SENP6,TPBG,NT5E,PM20D2,FOXO3,MARCKS,NUS1,GJA1,TPD52L1,HINT3,MYB,CCDC28A,PHACTR2,AL049844.1,UTRN,WTAP,IGF2R,QKI,GNA12,ACTB,RNF216,EIF2AK1,CYTH3,NDUFA4,SKAP2,HOXA11,JAZF1,GGCT,RP9,C7orf25,AC010132.3,NUDCD3,SBDS,POM121C,SEMA3C,CYP51A1,CDK6,PDK4,GIGYF1,EPHB4,NAPEPLD,NRCAM,PNPLA8,DOCK4,ZNF800,RBM28,PODXL,CHCHD3,SLC35B4,C7orf49,MTPN,CREB3L2,ZC3HAV1,SLC37A3,TMUB1,KMT2C,ESYT2,SUN1,EIF3B,UMAD1,GLCCI1,SP4,CCDC126,PRR15,GARS,AVL9,RALA,CCM2,CCT6A,SUMF2,CRCP,TYW1,BUD23,MDH2,CROT,PEG10,TRRAP,ZNF655,ZKSCAN1,AGFG2,SERPINE1,AP1S1,DLD,CAPZA2,ING3,METTL2B,CALU,KLHDC10,MKLN1,CALD1,NUP205,UBN2,ZNF783,ACTR3B,EN2,RBM33,NOM1,UBE3C,MFHAS1,CTSB,DLC1,PSD3,EGR3,PNMA2,SARAF,RAB11FIP1,FGFR1,IMPAD1,ASPH,ARMC1,PDE7A,JPH1,VIRMA,TP53INP1,STK3,RNF19A,ANKRD46,KLF10,AZIN1,TRPS1,TMEM65,WASHC5,FAM84B,FAM49B,EPPK1,ERI1,TNKS,MTMR9,FDFT1,ATP6V1B2,CHMP7,DOCK5,FZD3,LEPROTL1,MAK16,DDHD2,TACC1,GPAT4,VDAC3,MRPL15,LYN,CHD7,YTHDF3,CSPP1,ZBTB10,E2F5,SDC2,MTDH,SPAG1,FZD6,SQLE,ZNF623,C8orf33,NFIB,PSIP1,PLIN2,ELAVL2,SMU1,AQP3,NOL6,SIGMAR1,GNE,RNF38,ABHD17B,C9orf40,GNAQ,TLE1,UBQLN1,HNRNPK,AGTPBP1,GOLM1,GAS1,NOL8,IPPK,FAM120AOS,TRIM14,TMEM245,EPB41L4B,PTGR1,SUSD1,PTBP3,ALAD,POLE3,RAB14,STOM,STRBP,FAM129B,FAM102A,NACC2,TMEM250,ABCA2,KANK1,KDM4C,DNAJA1,CHMP5,DAPK1,SPIN1,FAM120A,MFSD14B,TGFBR1,NIPSNAP3A,PALM2-AKAP2,AKAP2,ATP6V1G1,NEK6,GAPVD1,ZBTB34,URM1,SET,GPR107,NCS1,COL5A1,EGFL7,PUDP,PIR,RPS6KA3,KLHL15,SMC1A,HUWE1,ZMYM3,RLIM,MORF4L2,RBM41,TSC22D3,ACSL4,AMOT,RPL39,UPF3B,ZDHHC9,RAP2C,STS,WWC3,TMSB4X,SMS,PRRG1,MID1IP1,USP9X,DDX3X,ZNF81,EBP,TBC1D25,UBQLN2,DLG3,NONO,OGT,CHIC1,PBDC1,TCEAL9,FAM199X,TMEM164,SLC6A14,UBE2A,ZBTB33,XIAP,FMR1,VMA21 |
